# Placement of Leucine Zipper Motifs at the Carboxyl Terminus of HIV-1 Protease Significantly Reduces Virion Production

**DOI:** 10.1371/journal.pone.0032845

**Published:** 2012-03-01

**Authors:** Yen-Yu Pan, Shiu-Mei Wang, Kuo-Jung Huang, Chien-Cheng Chiang, Chin-Tien Wang

**Affiliations:** 1 Department of Medical Research and Education, Taipei Veterans General Hospital, Taipei, Taiwan; 2 Institute of Clinical Medicine, School of Medicine, National Yang-Ming University, Taipei, Taiwan; University of South Florida, United States of America

## Abstract

Natural HIV-1 protease (PR) is homodimeric. Some researchers believe that interactions between HIV-1 Gag-Pol molecules trigger the activation of embedded PR (which mediates Gag and Gag-Pol cleavage), and that Gag-Pol assembly domains outside of PR may contribute to PR activation by influencing PR dimer interaction in a Gag-Pol context. To determine if the enhancement of PR dimer interaction facilitates PR activation, we placed single or tandem repeat leucine zippers (LZ) at the PR C-terminus, and looked for a correlation between enhanced Gag processing efficiency and increased Gag-PR-LZ multimerization capacity. We found significant reductions in virus-like particles (VLPs) produced by HIV-1 mutants, with LZ fused to the end of PR as a result of enhanced Gag cleavage efficiency. Since VLP production can be restored to wt levels following PR activity inhibition, this assembly defect is considered PR activity-dependent. We also found a correlation between the LZ enhancement effect on Gag cleavage and enhanced Gag-PR multimerization. The results suggest that PR dimer interactions facilitated by forced Gag-PR multimerization lead to premature Gag cleavage, likely a result of premature PR activation. Our conclusion is that placement of a heterologous dimerization domain downstream of PR enhances PR-mediated Gag cleavage efficiency, implying that structural conformation, rather than the primary sequence outside of PR, is a major determinant of HIV-1 PR activation.

## Introduction

Human immunodeficiency virus type 1 (HIV-1) *gag* encodes a polypeptide Pr55*^gag^* that can self-assemble into virus-like particles (VLPs) [1]. During or soon after virus release from cells, Pr55*^gag^* is cleaved by viral protease (PR) into four major products: matrix (MA, p17), capsid (CA, p24), nucleocapsid (NC, p7), and p6 domains [1]. PR is encoded by *pol*, which is initially translated as a Pr160*^gag-pol^* polyprotein by a ribosomal frameshift event that occurs at a frequency of 5%, resulting in the expression of Pr160*^gag-pol^* to Pr55*^gag^* at a ratio of approximately 1∶20 [2]. Pr160*^gag-pol^* is incorporated into virions via interactions with assembling Pr55*^gag^* [3], [4], [5], [6], [7], [8]. Pr160*^gag-pol^* cleavage by PR yields reverse transcriptase (RT) and integrase (IN) in addition to Gag products. The PR-mediated proteolytic cleavage of Pr55*^gag^* and Pr160*^gag-pol^*, known as virus maturation, is essential for the acquisition of viral infectivity [9], [10], [11], [12], [13].

How PR is activated to mediate virus maturation is not completely clear. One proposal is that interaction among Pr160*^gag-pol^* molecules triggers the activation of embedded PR, which in homodimeric form mediates Gag and Gag-Pol cleavage following PR autocleavage from Pr160*^gag-pol^*. Maintenance of the Pr55*^gag^*/Pr160*^gag-pol^* expression ratio is critical to virus assembly; the artificial overexpression of Pr160*^gag-pol^* or PR drastically reduces virion production as a result of enhanced Gag processing by overexpressed PR activity [14], [15], [16], [17], [18], [19], [20]. Equally important is the Pr160*^gag-pol^* sequence and structure, since sequence mutations upstream or downstream of PR often result in defective virus maturation or Gag cleavage [4], [21], [22], [23], [24]. Impaired Gag cleavage is assumed as being due, at least in part, to impaired PR activation, which is likely secondary to inadequate PR dimer interaction. Since natural RT is heterodimeric [25], [26], there is speculation that RT in the Gag-Pol context facilitates Pr160*^gag-pol^*-Pr160*^gag-pol^* interaction via RT-RT interaction, which in turn influences PR activation. Consistent with this scenario, RT deletion mutations can lead to severely impaired PR-mediated Gag processing [23]. In addition, efavirenz (EFV), a non-nucleoside reverse transcriptase inhibitor that enhances RT dimerization in vitro [27], [28], reduces virus production as a result of greatly enhanced Gag and Gag-Pol cleavage [29], [30]. Furthermore, a single amino acid substitution in RT (W402A) leads to significantly reduced virus production due to markedly enhanced PR-mediated Gag cleavage [31]. Combined, these data suggest that the RT domain plays an important role in PR activation by influencing PR dimer interaction.

It is likely that altered conformation induced by the RT mutation significantly impacts PR dimer interaction, resulting in premature or impaired PR activation. Accordingly, structural conformations rather than specific sequences may be major determinants of the PR activation process. A protein sequence unrelated to HIV-1 but possessing dimerization capacity may therefore promote PR activation by facilitating PR dimer interaction when fused to the end of PR. To test this possibility, we removed the RT and IN sequences and placed a leucine zipper (LZ)-coding sequence at the C-terminus of PR. Results indicate that LZ placement significantly reduced virion release due to enhanced Gag cleavage, similar to observations for RT W402A mutations. These results support the hypothesis that the placement of heterologous protein dimerization sequences downstream of PR can significantly enhance Gag processing efficiency by promoting PR activation.

## Results

### Placement of leucine zipper motifs at the C-terminus of PR results in significantly reduced virion production

**Figure 1:**
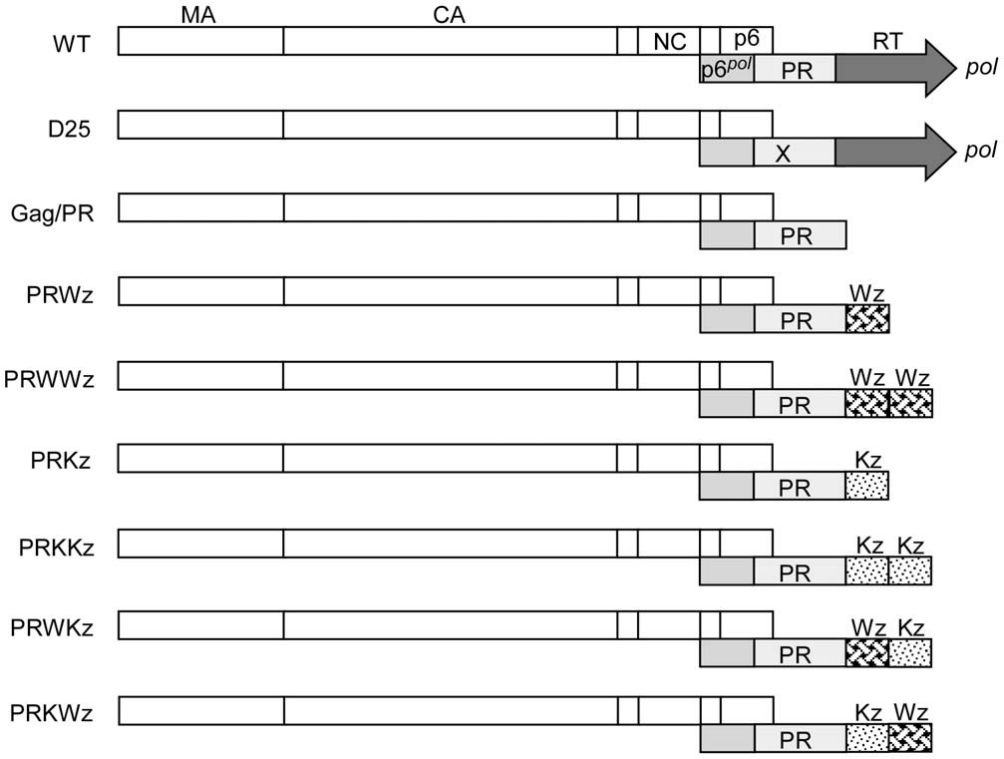
Schematic representations of HIV-1 Gag and Gag-PR-leucine zipper expression constructs. WT and D25 both expressed Pr55*^gag^* and Pr160*^gag-pol^*. Indicated are the HIV Gag protein domains MA (matrix), CA (capsid), NC (nucleocapsid), p6, *pol*-encoded p6*^pol^*, PR, and RT. The “X” in D25 denotes a PR-inactivated mutation. Gag/PR contains a stop codon insertion at the PR-RT junction, with a codon sequence of 5′-TTT CCC ATT AGC CCT TAG-3′ (RT codons underlined). Striped (Wz) and dotted (Kz) boxes denote wild-type (wt) and mutant (Kz) leucine zipper (LZ) domains, respectively, with each placed individually or in tandem repeat at the end of Gag/PR. Note that the chimeric constructs contain a linker of four Gly residues between the end of PR and beginning of LZ, and a separate linker of three Gly residues between the linked LZ domains.

**Figure 2:**
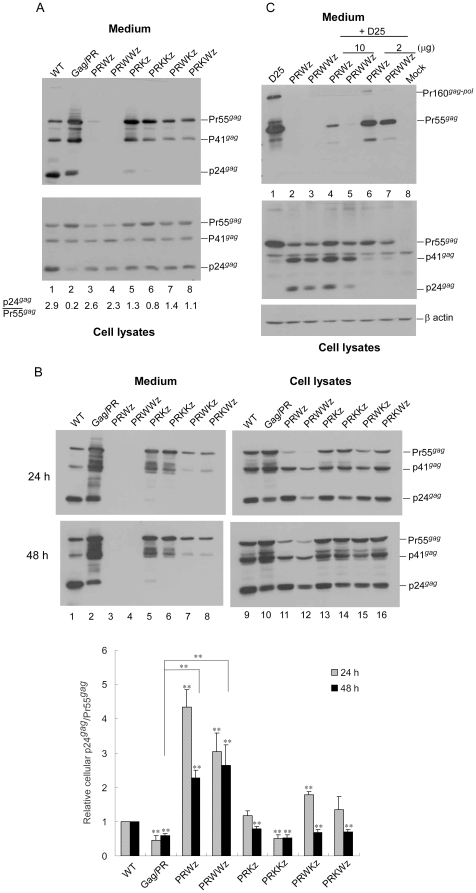
Effects of leucine zipper insertion on virus assembly and processing. (A–B) 293T cells were transfected with designated constructs Panel A: At 48–72 h, cells and culture supernatant were collected. Panel B: At 4 h post-transfection, equal amounts of cells were placed on two dish plates. Cells and culture supernatants were collected at 24 and 48 h post-transfection and subjected to Western immunoblotting. HIV-1 Gag proteins were probed with an anti-p24CA monoclonal antibody. Pr55*^gag^*, p41*^gag^*, and p24*^gag^* positions are indicated. Cellular Pr55*^gag^* and p24*^gag^* levels were quantified by scanning Pr55*^gag^* and p24*^gag^* band densities from immunoblots. Ratios of p24*^gag^* to p55*^gag^* were determined for the wt and each mutant (panel A, bottom), or normalized to those of wt in parallel experiments (panel B). Values were derived from three independent experiments. Bars indicate standard deviation. **, *p*<0.01. (C) 293T cells were transfected with 10 µg of D25, PRWz, or PRWWz plasmid alone (lanes 1–3); 10 µg D25 plus 10 µg PRWz (lane 4) or PRWWz (lane 5); or 10 µg D25 plus 2 µg PRWz (lane 6) or PRWWz (lane 7). For each transfection, plasmid DNA amounts were maintained at 20 µg by adding pBlueScript SK. At 48–72 h post-transfection, culture supernatant and cells were collected and subjected to Western immunoblotting.

To determine whether forced PR dimer interactions affect virus assembly and processing, we fused a LZ protein dimerization domain either singly or in tandem repeat to the C-terminus of an HIV-1 Gag-Pol truncated construct (Gag/PR), which is virus-assembly competent but processing-defective [23]. The resulting constructs were designated PRWz and PRWWz (Fig. 1). We used PRKz and PRKKz constructs containing the dimerization-defective LZ mutant version (Kz) as controls. Kz fusion to PRWz at the wt LZ C- and N-termini yielded constructs PRWKz and PRKWz, respectively. Each mutant was transiently expressed in 293T cells. Virus particle assembly and processing were analyzed by Western immunoblotting. The results shown in Figure 2A indicate that Gag/PR transfectants produced substantial quantities of VLPs at levels that were near wild-type. Unprocessed Gag (the Gag precursor Pr55) and incompletely processed Gag (the intermediate p41gag) represent two major Gag products compared to wt in our supernatant and cell samples (Fig. 2A, lanes 1 vs. 2). This is consistent with a report stating that a deletion in a downstream *pol* sequence significantly impairs PR-mediated virus maturation [23].

PRWz and PRWWz transfectants expressed readily detectable Gag, but produced barely detectable virus-associated Gag, suggesting a severe defect in virus assembly or release (Fig. 2A, lanes 3 and 4). In contrast, cells transfected with PRKz or PRKKz released readily detectable (though incompletely processed) Gag (lanes 5 and 6), similar to the Gag/PR scenario. VLP levels produced by PRWKz and PRKWz (lanes 7 and 8) were in-between those produced by PRWz and PRKz. Since enhanced or premature Gag cleavage by PR can lead to significantly reduced virus release, and since the wt LZ fusion-containing constructs exhibited higher ratios of cellular p24*^gag^* to Pr55*^gag^* compared to those found in Gag/PR cell lysates (Fig. 2A, lanes 3–4 vs. lane 2), we suggest that the LZ-associated virus production defect was largely due to enhanced Pr55*^gag^* cleavage efficiency.

Since cell samples were collected between 48 and 72 h post-transfection, it is possible that Gag processing reached a level of stability that prevented us from detecting any differences in efficiency between the wt form and mutants. To test this possibility, and to confirm the effect of LZ domain placement on Gag processing efficiency, we collected samples at 24 and 48 h following the transient expression of wt and mutants. We observed that both PRWz and PRWWz showed significantly higher cellular p24*^gag^*/Pr55*^gag^* ratios compared to those of wt or Gag/PR (Fig. 2B). Although PRWKz and PRKWz showed cellular Gag processing profiles similar to those of PRKz and PRKKz at 48 h, they displayed higher p24*^gag^*/Pr55*^gag^* ratios compared to Gag/PR at 24 h post-transfection (Fig. 2B upper panel, lanes 15–16 vs. lane 10). This suggests that LZ domain placement significantly enhanced Gag processing efficiency. The virus-associated Gag precursor that we detected may reflect, at least in part, the release of assembled Gag molecules that escaped PR-mediated cleavage (Fig. 2B, lanes 7–8).

To determine whether LZ placement affected virion production by wt or assembly-competent mutants *in trans*, we coexpressed PRWz or PRWWz with the wt or the HIV-1 protease-defective mutant D25, and observed that virus-associated Gag was markedly reduced when D25 was cotransfected with either PRWz or PRWWz at a 1∶1 ratio (Fig. 2C). Similar results were observed when the PRWz or PRWWz was coexpressed with a wt HIV-1 expression vector (data not shown). Combined, these results suggest that (a) PRWz and PRWWz both provided functional PR, and (b) the LZ-triggered virion assembly defect was primarily due to a higher Gag cleavage efficiency. The PRWWz transfectant frequently expressed a lower Gag level compared to other constructs (Fig. 2B, lane 12), likely the result of increased proteolytic degradation of Gag mediated by the PR.

### The PRWWz virus assembly defect is PR activity-dependent

**Figure 3:**
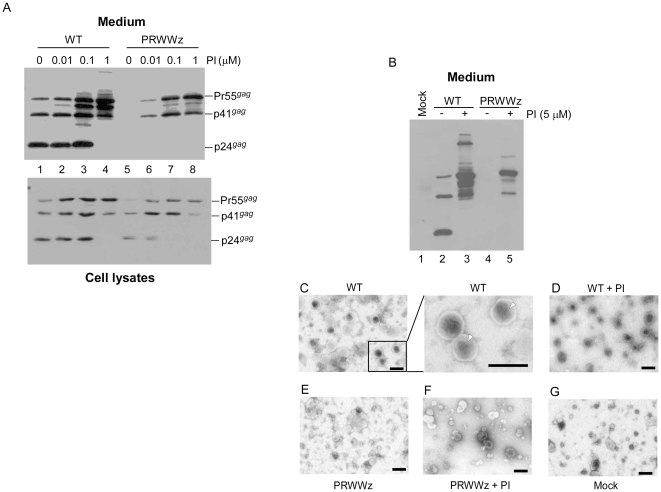
The PRWWz assembly defect is HIV-1 protease activity-dependent. (A) 293T cells were transfected with wt or mutant plasmids. At 4 h post-transfection, cells were replated on four dish plates and either left untreated (lanes 1 and 5) or treated with the HIV-1 protease inhibitor (PI) Saquinavier at concentrations of 0.01 (lanes 2 and 6), 0.1 (lanes 3 and 7), or 1.0 µM (lanes 4 and 8). At 48–72 h post-transfection, cells and culture supernatant were collected, prepared, and subjected to Western immunoblot analysis. (B–G) Transmission electron microscopy images of concentrated culture supernatant from 293T cells expressing the wt or PRWWz. Culture supernatants from PI-treated or untreated transfectants were collected, filtered, and pelleted through 20% sucrose cushions. Virus-containing pellets resuspended in PBS buffer were subjected to Western immunoblotting (panel B) and transmission electron microscopy. The high-power view (×60,000 magnification) in the inset shows cone-shaped cores (arrowheads) that are characteristic of mature wt virus particles (Panel C). Bars, 200 nm.

To determine whether the PRWz or PRWWz assembly defect is directly associated with viral PR activity, we treated PRWz and PRWWz transfectants with Saquinavier, an HIV-1 PR inhibitor (designated as PI). As expected, virus-associated PRWWz Gag (Pr55*^gag^* and p41*^gag^*) that was previously undetectable (or barely detectable) became readily detectable when PI concentrations were gradually increased (Fig. 3A). We noticed that wt cellular p24*^gag^* was still easily detected, even though PRWWz p24*^gag^* was undetectable under the same treatment conditions (Fig. 3A, lanes 3 vs. 7). This suggests that PRWWz is more susceptible to a protease inhibitor than wt, despite having higher Gag cleavage efficiency.

Since we centrifuged the culture supernatant through 20% sucrose cushions, we assumed that the recovered Gag would be present in pelleted particles. To confirm that the recovered Gag was from VLPs, we observed supernatant samples (Fig. 3B) with a transmission electron microscope, and found spherical wt and mutant Gag particles with electron-dense cores in PI-treated transfectant samples (Figs. 3D and 3F). However, mature virions with cone-shaped cores were only detected in non-PI-treated wt transfectant samples (Fig. 3C). Some vesicles lacking cores were noted, but virion-size particles containing electron-dense cores were not detected in mock-transfected samples, or barely detected in PRWWz transfectant supernatant that had not been treated with PI (Figs. 3E and 3G). These data support the hypothesis that the LZ-incurred assembly defect is PR activity-dependent.

### LZ enhancement of Gag cleavage is correlated with increased Gag-PR-LZ multimerization capacity

**Figure 4:**
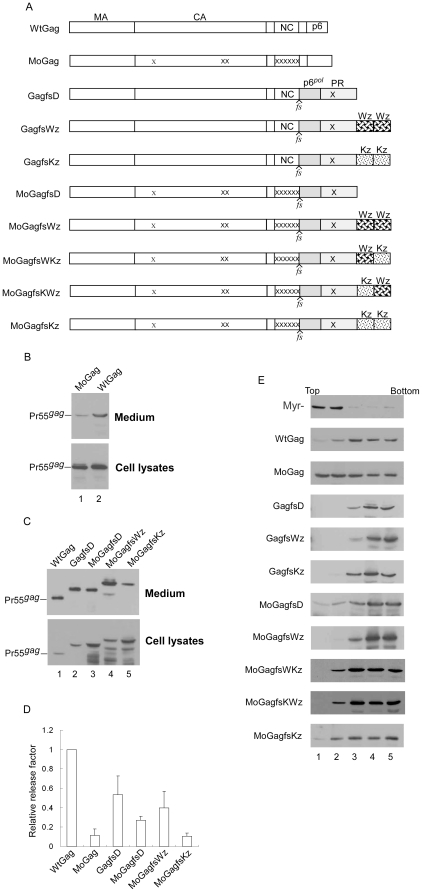
Effects of leucine zipper domain insertion on Gag-PR multimerization and assembly. (A) Schematic representations of HIV-1 Gag and Gag-LZ chimeras. HIV-1 Gag domains, *pol*-encoded PR, and the transframe peptide p6*^pol^* are indicated; *fs* denotes a frameshift mutation that forces *gag* and *pol* into the same reading frame. The fused leucine zipper (LZ) domains wt (Wz) or mutant (Kz) at the PR C-terminus are indicated as described in the Figure 1 legend; x denotes substitution mutations in CA, NC (NC15A), and PR that blocked either Gag assembly or PR activity. (B–E) Assembly and multimerization of HIV-1 Gag mutants. 293T cells were transfected with designated constructs. At 48–72 h post-transfection, culture supernatants and cells were collected and subjected to Western immunoblotting (panels B and C). (D) Gag-associated proteins from medium or cell samples were quantified by scanning immunoblot band densities (C). Ratios of Gag in media to Gag in cells were determined for each construct, and compared with wt release level by dividing the release ratio for each chimera by the wt ratio. (E) Velocity sedimentation analysis of cytoplasmic Gag precursor and Gag-LZ chimeras. Homogenized and extracted cytoplasmic lysates were centrifuged through consecutive 25%, 35%, and 45% sucrose gradients at 130,000×*g* for 1 hour. Fractions were collected from the top of each gradient. Aliquots of each fraction were subjected to SDS-PAGE (10%) and probed with a monoclonal antibody directed against HIV-1 CA.

We looked for correlations between enhanced PR-mediated Pr55*^gag^* cleavage efficiency and increased Gag-PR-LZ multimerization capacity. Believing that the potent Gag assembly domain might determine chimera multimerization status, we predicted that the contribution of LZ to enhanced chimera multimerization would be barely (if at all) detectable. We therefore assessed chimera multimerization capacity in a Gag assembly-defective context. After constructing an assembly-defective mutant (designated MoGag) and confirming that the mutation significantly impaired Gag assembly (Fig. 4B), we cloned PR-LZ chimeras into MoGag. To block the effect of PR activity on Gag-PR-LZ chimera assembly assays, all chimeras were introduced into a PR-inactivated HIV-1 Pr160*^gag-pol^*-expression plasmid GPfs [4], with *gag* and *pol* in the same reading frame (Fig. 4A). Results from repeated independent experiments indicate that chimeras with predicted molecular weights were detected in both supernatants and cell lysates following transient expression in 293T cells, suggesting that all chimeras were capable of assembly and release to some extent. However, we noted that MoGagfsWz transfectants produced more chimeric VLPs than MoGagfsD or MoGagfsKz (Figs. 4C and 4D). To confirm that MoGag was multimerization-defective and that LZ did enhance assembly-defective Gag multimerization, we subjected wt Gag and each mutant to velocity sedimentation analyses. A non-myristylated (myr-) Gag mutant [32] known to be severely defective in both membrane binding and multimerization served as a negative control. Our data indicate that most of the wt Gag was recovered at fractions 3 to 5; in contrast, most myr- Gag and substantial amounts of MoGag were recovered at fractions 1 and 2. Portions of MoGagfsD and MoGagfsKz were detected at lower sucrose density fractions, whereas MoGagfsWz was almost completely recovered at higher sucrose density fractions (Fig. 4E). Unlike MoGagfsWz, which mostly sedimented at fractions 4 and 5, considerable amounts of MoGagfsWKz and MoGagfsKWz were also recovered at fraction 3, and low but detectable amounts were observed in fraction 2. This sedimentation pattern was similar to that of MoGagfsKz (Fig. 4E, three bottom panels). Also similar to MoGagfsKz, both MoGagfsWKz and MoGagfsKWz were incapable of efficiently assembling into chimeric VLPs (data not shown). We observed this result in repeated independent experiments. This finding suggests that when fused to the PR C-terminus, a LZ tandem repeat containing a wt and mutant LZ (WKz or KWz) does not enhance Gag-PR multimerization as effectively as a wt LZ tandem repeat (Wz). This may partly explain why PRWKz and PRKWz showed relatively lower Gag cleavage efficiency compared to PRWWz (Fig. 2).

Compared to MoGag, which had significant amounts of Gag detected at lower sucrose density fractions (1 and 2), MoGagfsD molecules were mostly sedimented at fractions 3 to 5, a difference that may be explained in part by p6*^pol^*-PR contributing to MoGagfsD multimerization via PR dimer interaction. Although GagfsD presented an efficient multimerization profile, it produced VLPs at a relatively lower level compared to WtGag (Figs. 4C and 4D). This may have been due to its lack of p6*^gag^*, which is required for efficient virus budding [33], [34].

**Figure 5:**
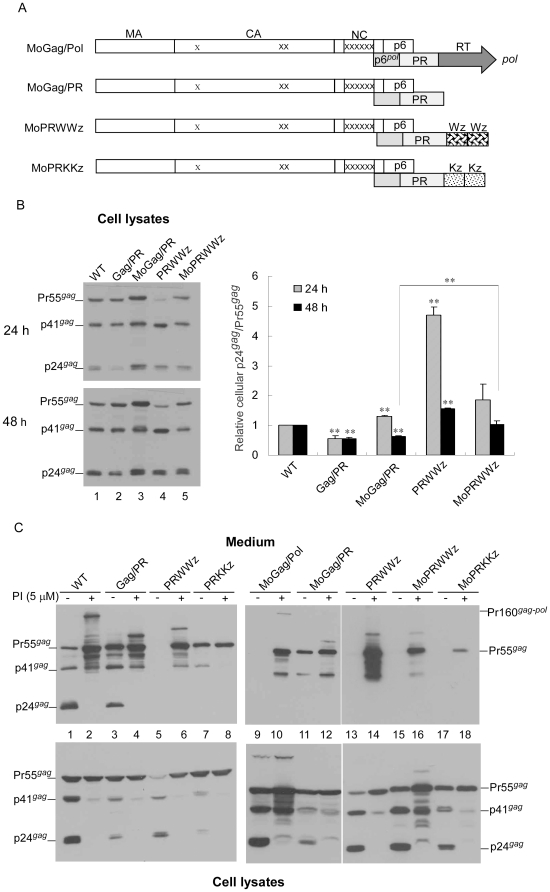
Effects of LZ domain insertion on Gag cleavage. (A) Schematic representations of HIV-1 mutants. Indicated are domains for HIV-1 Gag and *pol*-encoded p6*^pol^*, PR, and RT. Wild-type (Wz) and mutant (Kz) leucine zipper domains and “x” substitution mutations blocking Gag assembly are indicated as described above. (B–C) 293T cells were transfected with designated constructs. At 4 h post-transfection, equal amounts of cells were placed on two dish plates. Panel B: Cells were collected 24 and 48 h post-transfection and subjected to Western immunoblotting. Cellular Pr55*^gag^* and p24*^gag^* levels were quantified by scanning immunoblot band densities. Ratios of p24*^gag^* to p55*^gag^* were determined for each mutant and normalized to those of wt in parallel experiments. Bars indicate standard deviation. **, *p*<0.01. Panel C: Supernatants and cells were collected 48–72 h post-transfection following treatment with or without 5 µM of HIV-1 protease inhibitor Saquinavier. Samples were prepared and subjected to Western immunoblotting.

Our next question was whether LZ enhanced the cleavage efficiency of the assembly-defective mutant MoGag. MoGag with an LZ fusion was expressed in a Gag/PR context, thus expressing both Pr55*^gag^* and PR containing Gag-Pol or Gag-PR(-LZ) fusions (Fig. 5A). As expected, the insertion of LZ into MoGag/PR at the PR C-terminus resulted in significantly enhanced Gag cleavage efficiency (Fig. 5C, lanes 11 vs. 15), suggesting that the LZ enhancement of Gag-PR multimerization is correlated with increased PR-mediated Gag cleavage efficiency. Inefficient Gag cleavage, likely due to either impaired PR activation as a result of Pol truncation or to inhibited PR activity due to a protease inhibitor, resulted in improved MoGag VLP assembly (Fig. 5C, lanes 9–12 and 15–18). This suggests that the MoGag assembly defect is PR activity-dependent, at least in part. The MoPRWWz had significantly higher cellular p24*^gag^*/Pr55*^gag^* ratios compared to MoGag/PR, but slightly lower compared to PRWWz (Figs. 5B and 5C), suggesting that the multimerization-defective Gag mutation reduced the LZ-mediated enhancement of Gag cleavage. It also supports the proposal that the Gag assembly domain plays a role in PR activation.

**Figure 6:**
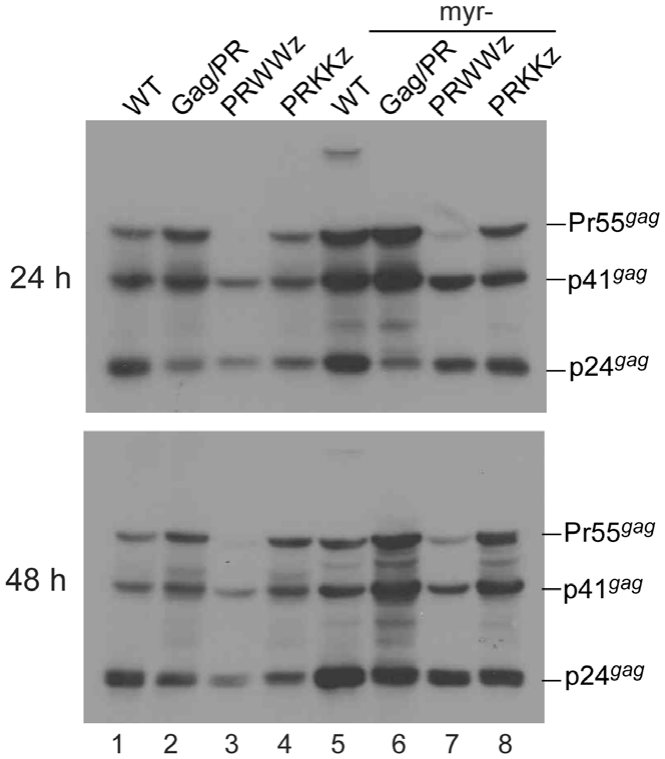
The enhancement effect of LZ on Gag cleavage is independent of myristylation. 293T cells were transfected with designated constructs containing a normal myristylation or myristylation-minus (myr-) mutation (lanes 5–8). At 4 h post-transfection, equal amounts of cells were placed on two dish plates. Cells and culture supernatants were collected at 24 and 48 h post-transfection and analyzed by Western immunoblotting.

Although myristylation is not required for Gag-Pol viral incorporation [7], [35], it is essential for Gag multimerization and virus assembly [11], [36]. To determine if a myristylation signal is necessary for the LZ enhancement effect on Gag cleavage, we introduced myr- into wt, Gag/PR, PRWWz, and PRKKz, and measured the Gag processing efficiency of each mutant. Our results indicate an efficient Gag processing profile for myr- PRWWz, similar to that of its myristylation-positive counterpart, PRWWz (Fig. 6). Since myristylation is essential to Gag membrane binding and virus assembly, we failed to detect virus-associated Gag products in any of the myr- supernatant samples (data not shown). This suggests that the LZ insertion made a significant contribution to enhanced Gag cleavage, regardless of the presence or absence of a myristylation signal.

## Discussion

Despite a lack of direct evidence, it is generally accepted that Gag-Pol molecule dimerization or multimerization triggers HIV-1 PR activation, which mediates Gag and Gag-Pol cleavage. Here we demonstrated that the insertion of LZ at the C-terminus of PR triggers markedly enhanced PR-mediated Gag cleavage efficiency, which is associated with increased Gag-PR multimerization capacity. This suggests that an HIV-1-unrelated protein sequence capable of self-association can enhance Gag cleavage efficiency when fused to the PR C-terminus. It also provides evidence in support of the assumption that Gag-Pol/Gag-Pol interaction triggers PR activation.

In an assembly-competent Gag/PR context, the wt LZ insertion resulted in markedly reduced, PR activity-dependent virus production (Fig. 2). Cellular Pr55*^gag^* was barely detected in PRWWz at 12 and 24 h post-transfection, while most Pr55*^gag^* remained unprocessed in wt transfectants 12 h post-transfection (data not shown). Combined, these results suggest that LZ-enhanced Gag cleavage is associated with premature Gag processing due to premature PR activation. We suggest that when fused at the PR C-terminus, the LZ domain facilitates PR-PR interaction via enhanced interactions among MA-CA-NC-PR-LZ chimeras. Gag is subsequently cleaved by PR *in trans*, either by PR embedded in the chimera, or by a mature and fully processed PR dimer.

Several researchers have suggested that the PR-mediated initial cleavage occurs via an intramolecular mechanism [37], [38], [39], [40], [41]. Although we did not search for the presence of a PR dimer, PR was theoretically capable of being released from the chimera as a fully processed dimer, since the cleavage site at the PR/LZ junction remained intact. Compared to the wt LZ fusion, the placement of a mutant LZ (Kz) at either the N- or C-terminus of the wt LZ (Wz) resulted in reduced Gag cleavage efficiency (Fig. 2). This may be attributable to the inability of WKz or KWz to promote Gag-PR multimerization (Fig. 4E).

Although myristylation is required for Gag membrane binding (which may in turn promote efficient Gag multimerization [32]), we found evidence that myr- Gag/Pol or MoGag/Pol were capable of mediating Pr55*^gag^* processing (Figs. 5 and 6). This finding suggests that neither membrane association nor an assembly-competent Gag domain is essential for the activation of PR embedded in Gag-Pol. It is likely that myr- Gag-Pol can still undergo dimerization to a level that is sufficient to trigger PR activation. Previous studies have shown that myr- Gag-Pol can efficiently cleave Pr55*^gag^ in trans* and be packaged into Pr55*^gag^* VLPs [4], [7], [35]. The multimerization defect as a result of membrane binding apparently does not significantly compromise the LZ enhancement of PR-mediated Gag cleavage.

The next question is why premature PR-mediated Gag and Gag-Pol cleavage do not occur during virus assembly, given that multiple assembly domains outside of protease promote Gag-Pol multimerization. One possibility is that the transframe peptide p6*^pol^* may play a role in modulating PR dimer interface interaction, thus preventing premature PR activation. Due to a blocking mutation at the p6*^pol^*/PR cleavage, p6*^pol^*-retaining PR is defective in mediating virus maturation [42], [43], [44], [45], suggesting that a fully functional PR requires the removal of p6*^pol^*. However, to our knowledge there are no reports on the role of p6*^pol^* in PR dimer interaction in a Gag-Pol context. We found that a Gag-Pol mutant with deleted p6*^pol^* was incapable of efficiently processing coexpressed Pr55*^gag^*, which argues against the possibility of p6*^pol^* playing a role in suppressing PR activation [46]. According to one recent study, the insertion of a larger reporter sequence in the p6*^pol^* region markedly impairs virus maturation, whereas partial substitution with a heterologous sequence does not [47]. This suggests that structural conformation, rather than a specific sequence upstream of PR, is important for determining PR activation.

Although the RT domain is essential for proper PR-mediated Gag cleavage, the RT homodimerization domain has no enhancement effect on Gag cleavage [48] unless EFV (an HIV-1 RT-dimerization enhancer) is added to culture medium [23], [49]. In contrast, we found that LZ is capable of triggering Gag cleavage enhancement when placed at the PR C-terminus. The RT domain apparently plays a role in preventing premature PR activation. Previous studies suggest that the downstream structural conformation of PR is important in terms of modulating PR activation. First, a single RT amino acid substitution (W402A) that is not known to have major impacts on *in vitro* RT dimerization [50] markedly enhances Gag processing [31]. An additional partial deletion at the C-terminus of p66RT not only reverses W402A-triggered Gag cleavage enhancement, but also markedly impairs virus maturation. In contrast, truncated Gag-Pol mutants that retain intact p66 or p51 RT domains still respond to the enhancement effect of W402A on Gag processing [31]. Second, substitution mutations in RT are capable of neutralizing the enhancement effect of EFV on Gag processing [49]. Combined, these data suggest that conformational changes in Gag-Pol may significantly affect PR dimer interface interactions, leading to either premature or insufficient PR activation. Our finding that the fusion of HIV-1-unrelated dimerization protein sequences at the C-terminus of PR sharply enhanced Gag cleavage strongly supports the hypothesis that structural conformation, rather than specific sequences, largely determines PR activation status. The RT structure domain in the Gag-Pol context may help prevent PR from premature activation via a conformation that prevents the PR dimer interface from interacting efficiently. This conformation may change during Gag-Pol packaging, thus supporting more efficient PR dimer interaction.

Pettit et al. [41] have demonstrated that inactivated PR dimer interface mutations can be compensated for to some extent by extra PR sequences in the Gag-Pol context. Altered PR dimerization kinetics or activity has been identified in several studies of PR-containing C- or N-terminal extensions into Gag-Pol [21], [23], [24], [38], [40], [43], [51], [52]. In most cases, it is not feasible to assay the impacts of these constructs on virus assembly or PR-mediated virus maturation, due to overlapping *gag* and *pol* reading frames. Our approach provides a convenient system for analyzing the impacts of mutations on PR dimer interactions by assessing virus particle assembly and processing. Studies are underway to determine if LZ sufficiently compensates for the inactivation of PR dimer interface mutations.

## Materials and Methods

### Plasmid construction

To place a leucine zipper (LZ) in frame into the HIV-1 PR C-terminus, we engineered a pBRCla-Sal plasmid cassette containing an HIV-1 coding sequence (from ClaI-nt.831 to SalI-nt.5786) and a pcDNA3.1-myc/hisA polylinker inserted at the IN C-terminus. ΔNC(wtZip) and ΔNC(Kzip) [53] served as templates for amplifying the respective wt and mutant LZ domains of human CREB [54] using the forward primer 5′-CGGGATCCTGGAGGAGGACGAGAGTGTCGTAG-AAAGAAG-3′ and reverse primer 5′-CCAAGCGGCCGCGATTTGTGGCAG-TAT-3′. Plasmids containing the wt and mutant LZ (provided by E. Barklis [55]) were used to construct the ΔNC(wtZip) and ΔNC(Kzip). The human CREB LZ sequence is 284-RECRRKKKEYVKCLENRVAVLENQNKTLIEELKALKDLYCHKSD-327. The underlined amino acid residues E298, R300, E305, Q307, I312, E314, and L321 were mutated to Lys, and N308 was mutated to His, yielding the mutant LZ [54]. Amplified fragments were digested with BamHI and NotI and ligated into the pBRCla-Sal cassette, yielding constructs PRWz and PRKz, respectively. To add the LZ copy, PCR-amplified wt and mutant LZ fragments were purified, digested with a restriction enzyme, and ligated into the PRWz and PRKz, yielding the constructs PRWWz, PRWKz, PRKKz, and PRKWz.

The Gag assembly-defective mutant MoGag was constructed by recombining the CA mutant M39A/W184A/M185A with NC mutant NC15A, which was kindly provided by P. Spearman [56]. NC15A has 15 NC-basic residues replaced with alanine. M39A/W184A/M185A was created by overlapping PCR with the following mutagenic primers: for M39A, 5′-CTGATAGCGCTGAAAATGCGGGTATCA-3′, and for W184A/M185A, 5′-CAACAACGTTTCTGTAGCCGCATTTTTTAC-3′. The MoGag mutation was cloned into the indicated PR-leucine zipper constructs. As described previously, Gag/PR has deleted RT and IN coding sequences [23]. In D25, Arg is substituted for the PR catalytic residue Asp [20]. The backbone of all expression constructs is the HIV-1 proviral plasmid HIVgpt [57].

### Cell culture and transfection

293T cells were maintained in DMEM supplemented with 10% fetal calf serum. Confluent 293T cells were trypsinized, split 1∶10 and seeded onto 10-cm dish plates 24 hours before transfection. For each construct, 293T cells were transfected with 20 µg of plasmid DNA by the calcium phosphate precipitation method (18), with the addition of 50 µM chloroquine to enhance transfection efficiency.

### Western immunoblot analysis

Culture media from transfected 293T cells were filtered through 0.45 µm-pore-size filters, followed by centrifugation through 2 ml of 20% sucrose in TSE (10 mM Tris-HCl pH 7.5, 100 mM NaCl, 1 mM EDTA) plus 0.1 mM phenylmethylsulfonyl fluoride [PMSF]) at 4°C for 40 min at 274,000×*g* (SW41 rotor at 40,000 rpm). Viral pellets then were suspended in IPB (20 mM Tris-HCl pH 7.5, 150 mM NaCl, 1 mM EDTA, 0.1% SDS, 0.5% sodium deoxycholate, 1% Triton X-100, 0.02% sodium azide) plus 0.1 mM PMSF. Cells were rinsed with ice-cold PBS (phosphate-buffered saline), scraped from the plates, collected in 1 ml of PBS and pelleted at 2,500 rpm for 5 min. The cell pellets were resuspended in 250 µl of IPB plus 0.1 mM PMSF, and then subjected to microcentrifugation at 4°C for 15 min at 13,700×*g* (14,000 r.p.m.) to remove cell debris. Either supernatant or cell sample was then mixed with equal volumes of 2× sample buffer (12.5 mM Tris-HCl pH 6.8, 2% SDS, 20% glycerol, 0.25% bromophenol blue) and 5% β-mercaptoethanol and boiled for 5 min. Samples were subjected to SDS-PAGE and electroblotted onto nitrocellulose membranes. Membrane-bound Gag proteins were immunodetected using an anti-p24*^gag^* (mouse hybridoma clone 183-H12-5C) monoclonal antibody at a 1∶5,000 dilution from ascites. The secondary antibody was a rabbit anti-mouse (HRP)-conjugated antibody at 1∶15,000 dilution as appropriate and the procedures used for HRP activity detection followed the manufacturer's protocol (Pierce). Immunodetected bands on film were quantified by using AlphaImager 2000 (Alpha Innotech Corp.) and Image J software.

### Velocity sedimentation analysis of cytoplasmic Gag proteins

Cells were rinsed twice with PBS, pelleted and resuspended in 1 ml TEN buffer containing Complete protease inhibitor cocktail followed by homogenization. The cell lysates then were centrifuged at 3,000 rpm for 20 min at 4°C. Five hundred µl of the postnuclear supernatants were mixed with an equal amount of TEN buffer, and were then applied to the top of a pre-made 25–45% discontinuous sucrose gradient. This gradient was prepared in TEN buffer containing 1 ml of each of 25%, 35%, and 45% sucrose. The gradient was then centrifuged at 130,000×*g* for 1 hour at 4°C. Five 0.8-ml fractions were collected from the top of the centrifuge tubes. The proteins present in aliquots of each fraction were precipitated with 10% TCA and subjected to western blot analysis as described in the membrane flotation assay.

### Electron microscopy

Virus-containing supernatants were centrifuged through 20% sucrose cushion. Concentrated viral sample was placed for 2 min onto a carbon-coated, UV-treated 200 mesh copper grid as described [58]. Sample-containing grids were rinsed 15 s in water, drained off water with filter paper, and stained for 1 min in filtered 1.3% uranyl acetate. Staining solution was drained off by applying filter paper to the edge of the grid. Grids were left to dry before viewing in a JOEL JEM-2000 EXII transmission electron microscope. Images were collected at 20,000× and 60,000×.
